# Glycosyltransferase-Mediated Exchange of Rare Microbial Sugars with Natural Products

**DOI:** 10.3389/fmicb.2016.01849

**Published:** 2016-11-16

**Authors:** Ramesh P. Pandey, Jae K. Sohng

**Affiliations:** ^1^Department of BT-Convergent Pharmaceutical Engineering, Sun Moon UniversityAsan-si, South Korea; ^2^Department of Life Science and Biochemical Engineering, Sun Moon UniversityAsan-si, South Korea

**Keywords:** glycosyltransferase, glycodiversification, glycosylation, natural products, rare microbial sugar

A large number of plant and microbial architectures have been identified and investigated for potential applications in therapeutics, cosmetics, and nutrition. Nonetheless, continuous identification, and design of novel lead molecules are prerequisites to tackling emerging diseases since most existing drugs are losing utility because of resistance by microorganisms. Advances in biotechnological tools in systems and synthetic biology, chemical biology and metabolic engineering, genome sequencing, and synthesis, protein engineering and mutagenesis have enabled alteration of the biological routes of natural product (NP) biosynthesis in heterologous robust hosts to produce a wide array of compounds (Pandey et al., 2016), thus adding diversity in the NPs. Post-modifications of NPs by tailoring enzymes is one of the promising approaches for engineering and manipulating NPs under human control with selective power. Glycosyltransferases (GTs) have been attracting tremendous attention because of their potential to diversify NPs by conjugating diverse types of sugar appendages (Williams et al., 2007, 2011; Pandey et al., 2014), and altering the physico-chemical and biological properties, such as adsorption, distribution, metabolism, and excretion of molecules (Weymouth-Wilson, 1997). For example, when mycosamine sugar was replaced by perosamine in amphotericin B, antifungal and hemolytic activities were improved in new derivative which has minimal inhibitory activity concentration (MIC) of 1.9 μg/ml compared to 2.1 μg/ml of amphotericin B against *Saccharomyces cerevisiae* (Hutchinson et al., 2010). Similarly, when D-desosamine of YC-17 was replaced with four sugars D-quinovose, L-rhamnose, L-olivose, and D-boivinose, the L-rhamnose sugar conjugated derivative exhibited better antibacterial activity than the parent YC-17 against erythromycin-susceptible and resistant *Enterococcus faecium* and *Staphylococcus aureus* (Shinde et al., 2013). GTs have also been exploited to reverse the glycosylation reactions in NPs (Zhang et al., 2006). This property of GTs expanded the possibility of synthesizing diverse nucleotide diphosphate (NDP)-sugars and exchanging them among different classes of NPs by single vessel trans-glycosylation (Zhang et al., 2014).

The application of GTs in the industrial biosynthesis of NP glycosides (De Bruyn et al., 2015), detoxification of pollutants, pesticides, and xenobiotics (Stupp et al., 2013), and homeostasis of plant hormones to control crop engineering (Tiwari et al., 2016) has profound impact on human daily life. Thus, plant and microbial GTs with broad substrate promiscuity have been identified and characterized to glycodiversify NPs to further widen their scope for the generation of future molecules for human use (Elshahawi et al., 2015; Tiwari et al., 2016). Such powerful enzymes can be engaged for the exchange of diverse sugar moieties using microbial cells engineered to produce secondary metabolites using metabolic engineering tools.

Most of the plant glycosylated secondary metabolites contain unmodified simple sugars, such as D-glucose, D-galactose, D-glucuronic acid, L-rhamnose, D-xylose, and D-arabinose and many more. Thus, plant GTs are usually studied for their catalytic potential to harness the aforementioned simple sugar moieties for a diverse class of metabolites, such as polyphenols, terpenoids, benzoates, salicyclic acids, and polyketides. In contrast to plant glycosides, microbial originated glycosylated molecules contain highly modified deoxysugars with different functional moieties, such as amino-, nitro-, keto-, and sulfo-groups in their structures. These modified sugar moieties are found to play a crucial role in executing biological functions to those microbial metabolites, such as doxorubicin, staurosporine, vancomycin, calicheamicin, amphotericin B, tylosin, and erythromycin (Kren and Rezanka, 2008).

GT-mediated exchange of sugar moieties of microbial origin with secondary metabolites in metabolically engineered hosts is a fascinating viable approach for the generation of novel biologically potent compounds (Chen, 2011). This approach can be achieved in two different ways as shown in Figure 1. The first approach is to supplement aglycone along with a chemically modified synthesized sugar in an engineered microbial cell harboring anomeric kinase (AK), nucleotidyl transferase (NT), and a promiscuous GT. All three enzymes should be capable of harnessing diverse sets of sugars, sugar phosphates, and NDP-sugars, respectively. AK phosphorylates exogenously supplemented sugar to sugar-1-phosphate, which is subsequently utilized by NT to produce NDP-sugar at an expense of NTP. Thus, the generated NDP-sugar is utilized by GT as a donor substrate and the sugar moiety is transferred to exogenously supplemented aglycone/acceptor molecules. The newly generated glycosides are usually excreted outside of the cell into the culture medium and are readily extracted and purified. In contrast to the first approach, the second *in vivo* glycosylation platform does not require the supplementation of chemically synthesized sugars. Nevertheless, rare NDP-sugars are produced in the cell cytosol by heterologous expression of diverse sets of sugar cassettes. These rare sugars are finally transferred to exogenously supplemented aglycones by substrate promiscuous GT. The production of diverse NDP-sugars in the cell cytosol by both approaches glycodiversify the exogenously added molecule.

**Figure 1:**
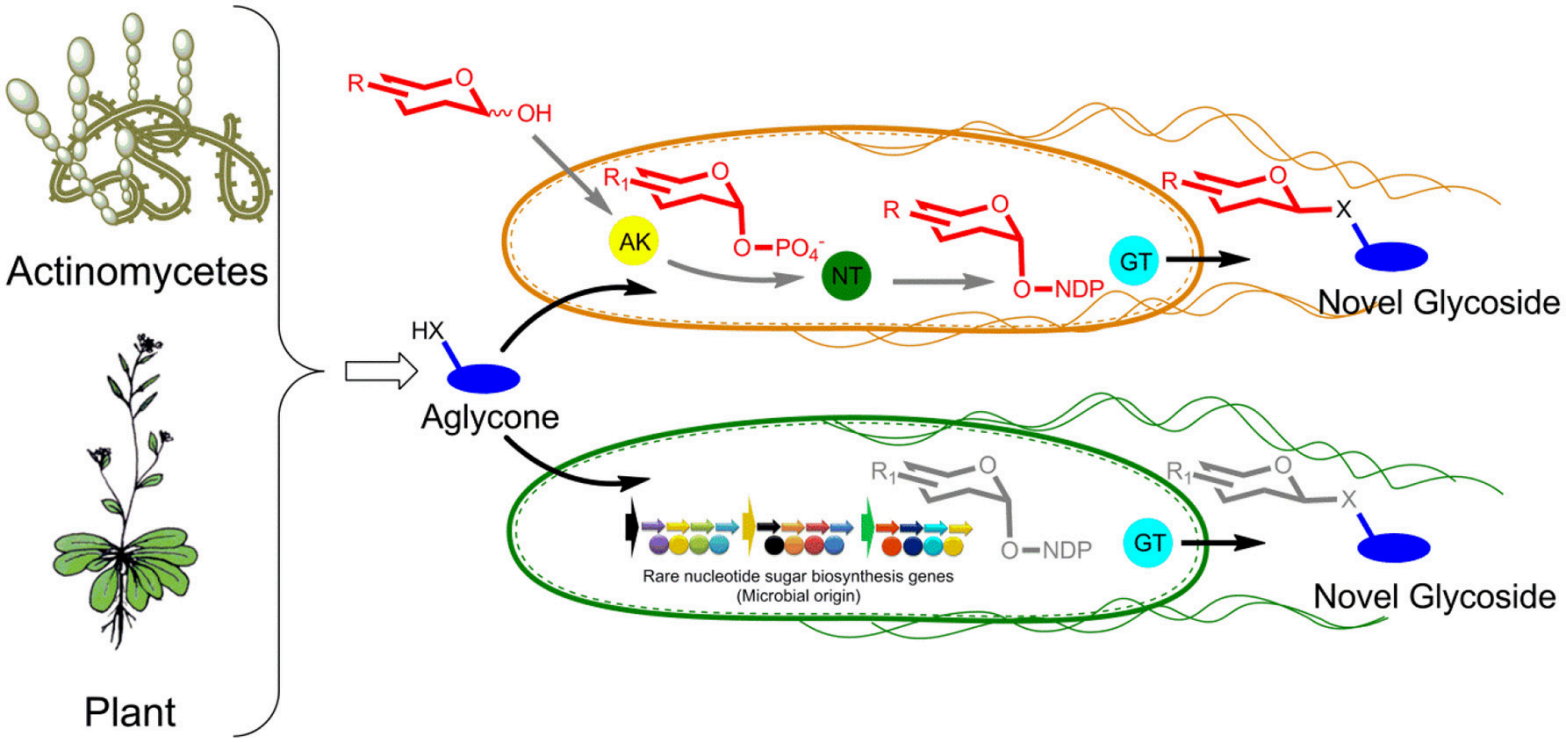
**GT-mediated microbial glycone and aglycone exchange platforms for NPs diversification by glycosylation (X = *O*, *S*, or *N*H)**. In first strain, reducing sugars and aglycones are fed while in second strain only aglycones are fed. AK, anomeric kinase; NT, nucleotidyl transferase; GT, glycosyltransferase.

Such green approaches for the *in vivo* glycodiversification of aglycones by diverse sugar appendages are superior to existing *in vitro* strategies. The necessity of multiple enzyme production and purification for enzymatic synthesis of NDP-sugars, low yield of purified target NDP-sugars, cofactor addition or regeneration during the process of reaction usually increases the process cost for *in vitro* glycorandomization (Fu et al., 2004). On the other hand, the chemical approach for NP glycosides synthesis is tedious, hazardous, time consuming, and requires protection and de-protection of the functional groups. Since there is a high chance of by-products formation, the purification of molecules is difficult, which ultimately lowers the production yield of the target molecule. Thus, the microbial approach of conjugating diverse sugars to aglycones is the simplest, cheapest, and easiest approach that can be readily scaled up to industrial level by simply fermenting engineered microbes and supplementing aglycones and sugars as required (Song et al., 2013). However, *in vivo* glycorandomization approach needs further development because some of the aglycone substrates fed to the microbial culture system require specific transporters for entry and exit of the substrates and products through the membrane, respectively. This problem of substrate transportation could be addressed by overexpression of specific transporter proteins to the host organisms. Additionally, endogenous NDP-sugars or free monosaccharides (first approach Figure 1) compete with target unnatural sugar that has to be transferred to aglycone during *in vivo* glycosylation reaction. Rewiring of endogenous sugar pathways in host cell by metabolic engineering could overcome this problem of substrates competition in the cell cytosol.

Simple and high-throughput screening methods are prerequisite to identify sugar-exchanged products and to access the activities of engineered GTs in glycosylation reactions. However, very few such analytical methods are developed to detect glycosylated products at relatively low concentration. Some of the high-throughput methods used in glycosylation reactions are (i) use of fluorescent compounds as acceptor substrates whose fluorescence signal decreases upon sugar conjugation (Gantt et al., 2008), (ii) fluorescence-activated cell sorting (FACS) analysis used to identify cells in which fluorescent labeled sugars conjugated molecules are entrapped (Yang et al., 2010), (iii) enzyme-linked immunosorbent assay (ELISA) based detection of glycosylated products using carbohydrate-binding proteins (Hancock et al., 2009), and (iv) pH based color change of indicator molecule in the course of glycosylation reaction (Park et al., 2009). Besides these methods, the most abundantly used analytical approach for glycosylation reactions is high-performance liquid chromatography (HPLC). HPLC could be coupled with high resolution mass spectrometry (HR/MS) for direct identification of the NP-glycosides. HPLC based analyses of glycosylation reactions are not applicable for high-throughput screening of large number of GTs and their mutants. Hence, the development of a more efficient high-throughput screening approach for GT-mediated glycosylation reactions is obligatory.

Recently, advances have been made in the construction of robust microbial cells for the synthesis of several biomolecules in *E. coli, S. cerevisiae*, and *Streptomyces*. These biosynthesis approaches include *de novo* pathway engineering, central carbon flux redirection for co-factor or precursor synthesis, the selection of alternative enzymes, protein engineering/mutagenesis, precursor directed mutasynthesis, modular metabolic engineering, system and synthetic biology tools, such as codon optimization, vector, promoter, ribosome binding site engineering, and the engineering and manipulation of other non-protein-coding sequences (Pandey et al., 2016). For microbial production of NP glycosides, though different enzymes sources, particularly GTs, are used, no system/synthetic biology tools are applied for co-factor regeneration in microbial cell cytosol. Flux analysis for co-factor regeneration and optimum cell growth maintenance is crucial for efficient biosynthesis of glycosides. Several pathway genes for rare NDP-sugars, particularly thymidine diphosphate (TDP)-sugars, can be heterologously expressed for the modification of TDP-4-keto-6-deoxy-D-glucose (TKDG), which is the major intermediate of TDP-deoxysugars (Thibodeaux et al., 2008). Fine tuning of all possible heterologously expressed genes is essential for the balanced production of final NDP-sugars. In most cases for the biosynthesis of non-natural glycosides, the major limiting factor is the production of NDP-sugar in the cell cytosol of *E. coli*. This hurdle can be addressed by regulating intermediate consuming pathway genes in the chromosome of host organisms. Several studies have been reported by our group (Malla et al., 2013; Pandey et al., 2015) and other researchers to block alternate biosynthesis pathway genes in *E. coli* by constructing background *E. coli* mutants (Kim et al., 2015). However, the complete knock-down of essential genes for cell wall biosynthesis hampers cell growth and necessitates additional precursors to supplement the intermediates of blocked pathways. Thus, to rule out these problems, we should apply recently developed gene regulation tools, such as clustered regularly interspaced short palindromic repeats interference (CRISPERi) (Larson et al., 2013) and sRNA mediated gene regulation systems (Yoo et al., 2013) to control the flow of NDP-sugar biosynthesis intermediates to undesired products without hampering cell health. Moreover, application of these tools can also maintain high cell density during fermentation. Beside these options, the adequate expression of heterologous genes in the engineered host is another important aspect for rare NDP-sugar biosynthesis. To balance the fine tuning of all gene expression, modular and multivariate NDP-sugar biosynthesis pathways should be constructed to address the accumulation of an intermediate NDP-sugar in the biosynthesis pathway. Besides the biosynthesis of rare NDP-sugars in the microbial cell cytosol, we also need to focus on GT engineering for the generation of promiscuous GT that can accept diverse sets of NDP-sugars and acceptors for forward glycosylation reactions. Only a few GTs have been studied in detail for broad applications to NP glycodiversification. This is limited because of the lack of sufficient GTs with crystal structures. Thus, further study should be focused on the screening of highly promiscuous GTs and their design/engineering based on crystal structure and computer based modeling approaches, saturation mutagenesis after identification of “hot spots” for directed evolution. Such engineered mutant GTs would have high potential to generate a wide array of glycodiversified NPs. Eventually upon the biosynthesis of a library of novel NP glycosides, those molecules could be accessed for possible applications in therapeutics, cosmetics, and nutraceutics.

The *in vivo* fermentation approach for producing NP glycosides is inadequate for producing a significant amount of glycosides by simple fermentation even though the process can be easily subjected to industrial scale up. To produce future NP glycosides by fermentation, a robust system should be developed that is capable of producing diverse NDP-sugars in the cell cytosol while engineering promiscuous GTs for sugar transfer to acceptor molecules. High level production of target non-natural NP glycosides can be achieved by simple microbial fermentation upon proper implementation of recently developed system/synthetic biology tools for the engineering of microbial host cells. Cumulatively, this approach of GT-mediated exchange of microbial non-natural glycones with exogenous aglycones offers huge combinatorial potential for the biosynthesis of novel future molecules for human use.

## Author contributions

RP and JS wrote the manuscript.

### Conflict of interest statement

The authors declare that the research was conducted in the absence of any commercial or financial relationships that could be construed as a potential conflict of interest.
